# Exploring the effect of human and animal population growth on vector-borne disease transmission with an agent-based model of Rhodesian human African trypanosomiasis in eastern province, Zambia

**DOI:** 10.1371/journal.pntd.0006905

**Published:** 2018-11-08

**Authors:** Simon Alderton, Ewan T. Macleod, Neil E. Anderson, Noreen Machila, Martin Simuunza, Susan C. Welburn, Peter M. Atkinson

**Affiliations:** 1 Lancaster Environment Centre, Lancaster University, Lancaster, United Kingdom; 2 Centre for Health Informatics, Computing and Statistics (CHICAS), Lancaster Medical School, Lancaster University, Lancaster, United Kingdom; 3 Geography and Environment, Faculty of Social and Human Sciences, University of Southampton, Southampton, United Kingdom; 4 Division of Infection and Pathway Medicine, Deanery of Biomedical Sciences, College of Medicine and Veterinary Medicine, 1 George Square, Edinburgh, United Kingdom; 5 The Royal (Dick) School of Veterinary Studies and the Roslin Institute, University of Edinburgh, Roslin, United Kingdom; 6 Department of Disease Control, School of Veterinary Medicine, University of Zambia, Lusaka, Zambia; 7 School of Geography, Archaeology and Palaeoecology, Queen's University Belfast, Northern Ireland, United Kingdom; 8 State Key Laboratory of Resources and Environmental Information System, Institute of Geographic Sciences and Natural Resources Research, Chinese Academy of Sciences, Beijing, China; Imperial College London, Faculty of Medicine, School of Public Health, UNITED KINGDOM

## Abstract

This paper presents the development of an agent-based model (ABM) to investigate *Trypanosoma brucei rhodesiense* human African trypanosomiasis (rHAT) disease transmission. The ABM model, fitted at a fine spatial scale, was used to explore the impact of a growing host population on the spread of disease along a 75 km transect in the Luangwa Valley, Zambia. The model was used to gain a greater understanding of how increases in human and domestic animal population could impact the contact network between vector and host, the subsequent transmission patterns, and disease incidence outcomes in the region. Modelled incidence rates showed increases in rHAT transmission in both humans and cattle. The primary demographic attribution of infection switched dramatically from young children of both sexes attending school, to adult women performing activities with shorter but more frequent trips, such as water and firewood collection, with men more protected due to the presence of cattle in their routines. The interpretation of model output provides a plausible insight into both population development and disease transmission in the near future in the region and such techniques could aid well-targeted mitigation strategies in the future.

## Author summary

African trypanosomiasis is a parasitic disease which affects humans and other animals in 36 sub-Saharan African countries. The disease is transmitted by the tsetse fly, and the human form of the disease is known as sleeping sickness. With human and animal populations growing across Africa, demand for space to settle is on the rise, and people are being forced to occupy increasingly marginal spaces. This behaviour has the potential to increase exposure to pre-existing biological hazards, including vector-borne diseases. This investigation utilises agent-based modelling techniques to investigate the implications of a growing and spreading human and animal population in a region affected by Rhodesian human African trypanosomiasis. The model incorporates previously developed spatial data for the Luangwa Valley case study in Zambia, along with demographic data for its current inhabitants, and a detailed, seasonally-driven tsetse lifecycle. Tsetse and potential human and animal hosts are modelled at the individual level, allowing each contact and infection to be recorded through time. By modelling at a fine-scale, we can incorporate detailed mechanisms for tsetse birth, feeding, reproduction and death, as well as a realistic theoretical human and domestic animal population increase, before considering the possible spatial and demographic impact.

## Introduction

Human African trypanosomiasis (HAT), also known as sleeping sickness, is a neglected tropical disease (NTD) [1], one of a group of the most common conditions which affect the poorest 500 million people living in sub-Saharan Africa [2]. HAT is a vector-borne, parasitic disease which is caused by two sub-species of the protozoan parasite *Trypanosoma brucei* s.l.: *T*. *b*. *rhodesiense* in eastern and southern Africa, and *T*. *b*. *gambiense* in West Africa [3]. HAT is transmitted cyclically by the tsetse fly (genus: *Glossina*), in which it undergoes a complex life-cycle [4]. *T*. *b*. *rhodesiense* HAT (rHAT) presents in humans as an acute disease [5] and, being a zoonosis, has implications for a wide range of wildlife [6,7] and domestic animals [8]. When present in animal reservoirs, usually in combination with several other trypanosomes which affect the health of livestock, the disease is referred to as African animal trypanosomiasis (AAT), considered to be an important factor in restricting economic development in Africa [9].

With a current fertility rate of 5 births per woman [10], the national population of Zambia was observed as approximately 14 million in 2010, and predicted to reach 43 million by 2050, and 105 million by 2100 [11]. As a result, population growth in Zambia has been highlighted as an area in need of control [12]. Domestic cattle play an important role in the transmission cycle of rHAT, providing a reservoir for infection, and an attractive and complacent feed target for the tsetse vector [13]. In 1999, it was estimated that cattle numbers in tsetse infested areas across Africa could be in the region of 47.5 million [14]. Through the use of a series of predictive equations, the Program Against African Trypanosomiasis (PAAT) information system suggested that this number could rise by 50 million head of cattle [15], and with some cattle populations observed to grow at a rate of 1.4% or higher, this growth could be achievable within 50 years [16].

Traditionally, epidemiological research has analysed human migration at a comparatively coarse spatial resolution, considering the movement of susceptible host populations into high risk areas, or the introduction of infected hosts into susceptible populations, as a means of explaining disease spread [17]. Examples of such approaches can be found for HIV (e.g. [18]) and measles (e.g. [19]). However, for vector-borne diseases, heterogeneity in the contact patterns between vectors and hosts is frequently observed (e.g. [20–22]) and, as a result, an understanding of how this variability can amplify (e.g. [23,24]), or dampen (e.g. [25]), transmission rates could be of particular use in targeting disease surveillance or prevention programmes.

On one hand, influxes of potential rHAT hosts can provide a plentiful supply of possible bloodmeals in a tsetse-rich region, especially if a large proportion of those hosts are cattle. However, if the migration coincides with the development of previously uninhabited areas, the implications for rHAT transmission are less clear [26]. For example, where anthropogenic development reduces the biodiversity of wildlife hosts in a region, the risk of human infection increases by providing an alternative feed source for the vector (e.g. [27]). However, similar anthropogenic development can disturb vector habitats, with one study suggesting that a population density of between 15–39 people per square kilometre would be sufficient to trigger a decline in a *Glossina morsitans* population [28,29].

Agent-based modelling (ABM) provides a means of simulating disease transmission in a spatially explicit way and incorporating factors which are often overlooked, such as human behaviour, activity-based movement, the density and mobility of the disease vectors, and the influence of additional hosts [30]. An ABM is a useful method of integrating a wide range of knowledge about a disease transmission system, including the setting of simple rules controlling each *individual* (e.g., including humans, livestock, wildlife and flies), with the bottom-up ‘generative’ simulation phase facilitating the creation of outcomes about the macroscopic behaviour and interactions of the *population* of agents [31]. Through the use of geographical information in epidemiological models, the impacts of land-use and land cover change can be incorporated, including landscape features which largely control the connectivity between hosts and vector habitats, inhibit movement, and ultimately modify infection and disease risk [32]. Such an ABM approach allows the construction of pathogenic landscapes and the recording of variation in the timing, location and probability of infection as a result of the landscape constraint on possible contact patterns, and can subsequently provide an increased knowledge of transmission cycles, and early warning of increased transmission risk [30] [33] [34].

The ability to test “what-if?” scenarios that could be used to aid policy-making or management decisions [35], or increase understanding of a transmission system [36], is made possible with ABMs. This paper investigates the evolving epidemiological landscape as a result of potential changes to host density and distribution (by comparing to a plausible current ABM representation of rHAT transmission in the region), building on investigation of human movement [37], transmission in the absence of seasonality [38], and a seasonally dependent representation of the disease system [39]. These perturbations are in the form of a population growth scenario in the study site in Eastern Province, Zambia in which human and cattle populations have increased, and are increasing, on previously undeveloped land. We investigate what impact this incremental growth in developed areas of the study region, along with the associated, proportional growth in human and domestic animal population, has on the spread of rHAT. Using an ABM approach, we consider if the change in population is likely to cause an increase in host exposure to the tsetse, or if the concurrent removal of suitable tsetse habitat will create a spatial disconnect, reducing the infection risk for those previously exposed. Furthermore, we explore whether the pattern of infection will be affected by changes in the density, distribution, age-structure and activities of the human population.

## Methods

### Study area

**Fig 1:**
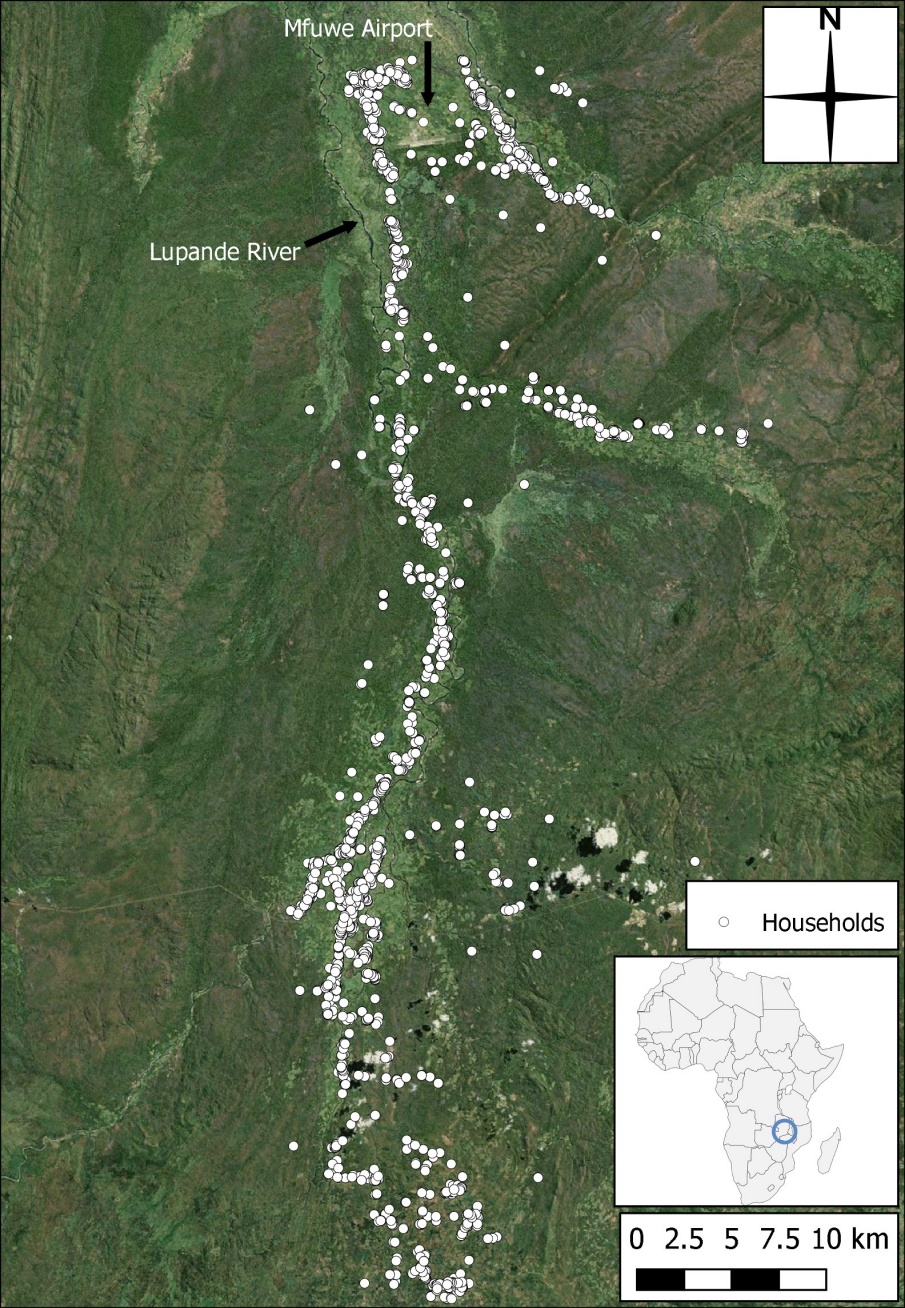
Map of the study area, showing size and location. Households from the census included in this modelling study are indicated as white circles, with Mfuwe airport highlighted in the north (produced using Landsat 7 imagery from USGS).

Eastern Province, Zambia is situated in southern Africa, sharing borders with Malawi (to the East) and Mozambique (to the South). The Luangwa Valley is an extension of the Great Rift Valley of East Africa, traversing the Zambian Eastern, Northern and Muchinga Provinces. The valley is flat bottomed and bounded by steep, dissected escarpments which rise to a plateau at approximately 900–1000 m [40]. The survey area and region to be modelled centres on a 75 km transect which starts close to Mfuwe Airport in the north, and runs southwards along the Lupande River and its distributaries (Fig 1). Villages in the study area are small (usually between five and 20 households) and the inhabitants are predominantly subsistence farmers.

Although the Luangwa Valley has had limited domestic livestock, with relatively consistent land-use activities over the last century centering around subsistence and semi-commercial agriculture [26,41], an influx of human population from the densely populated plateau region of Eastern Province into Mambwe, Katete and Nyimba Districts has been observed in recent decades, bringing new farming practices [42]. The Eastern Province study area in Zambia is no exception to the predicted rise in human and animal population in Zambia, with the encouragement of immigration in the last few decades bringing new farming techniques, and an increase in the livestock kept by new inhabitants [26]. Migrants have introduced cattle to the mid-Luangwa Valley as traction to support the intensive growing of cotton as a cash crop. The increasing numbers of cattle, goats and pigs which are now being kept in the region challenges previous beliefs that livestock keeping is not possible as a result of predation, disease prevalence and lack of veterinary support, and provides a new and constantly developing interface between the rHAT vector and hosts [26].

rHAT is endemic in the Luangwa Valley, first being observed in 1908 [43]. Today, cases of rHAT continue to be reported in the Luangwa Valley in small numbers (typically <10 cases per year); however, the true prevalence is unknown due to a poor understanding of the disease, social stigma, under-detection and under-reporting [26,44,45]. Land pressure associated with increasing levels of migration has resulted in human settlement in increasingly marginal, tsetse-abundant areas, previously avoided for fear of disease risk to introduced livestock. The described anthropogenic changes have the potential to destabilise current trypanosomiasis transmission cycles, with fear of the spread of rHAT into previously unaffected areas, and increasing prevalence of trypanosomiasis in both human and animal hosts: cattle are efficient reservoir hosts for rHAT in the absence of wildlife [46,47], and epidemics of AAT are more likely at new interfaces between humans, livestock and wildlife [48].

### ABM construction

This paper describes the application of an ABM for rHAT/AAT to a real world question; the effect of population growth, land conversion and settlement on rHAT risk in Eastern Province Zambia. The ABM was constructed using data derived from detailed rHAT, AAT, and tsetse ecological surveys, undertaken in 2013, in Eastern Province, Zambia [38,39]. The ABM model is complex, being constructed at fine spatial and temporal resolutions and incorporating the representation of multiple mechanisms (e.g. tsetse reproduction, tsetse feeding, human agent movements using real-world routines and pathfinding techniques [37]). A detailed description of the model framework, and the data used to construct it, are provided in [38], [39]. Therefore, only new data and modifications to the original model are described in detail here.

### Simulating population growth in the Luangwa Valley

For the baseline investigation, a human and animal census of the study area produced demographic data for 16,024 human inhabitants, 2,925 cattle, and 11,576 other domestic animals (including goats, pigs, donkeys, cats and dogs). Census data included age, gender, tribe and cattle ownership throughout the study region, together with the number and distribution of cattle and other domestic animals per household. Information was processed to determine human and animal agent distributions across the villages previously digitised in the study area.

**Fig 2:**
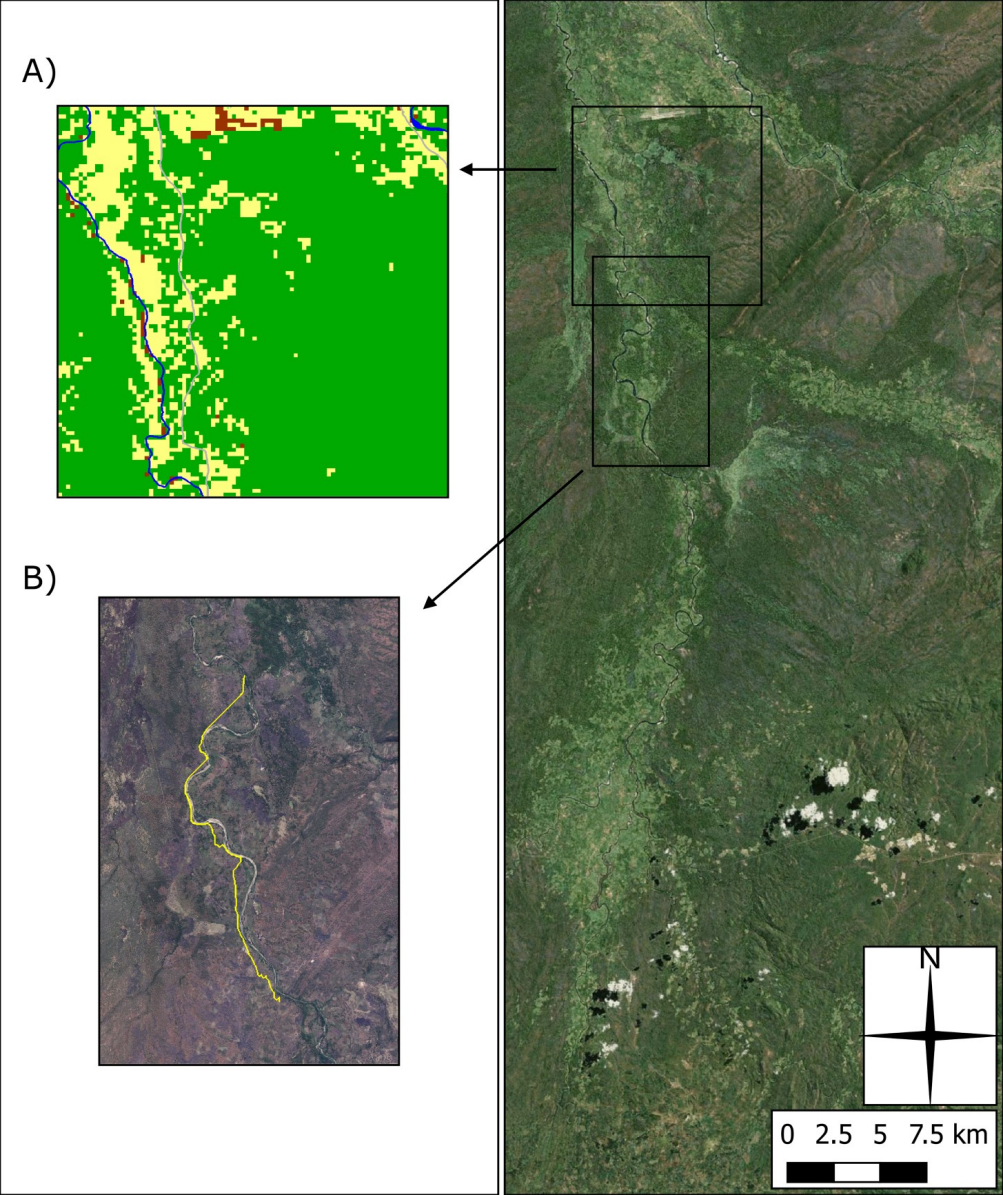
(A) A section of the 11 m spatial resolution land classification image, illustrating bare land areas around the airport (brown), cropland (yellow), bush/forest (green), and finer scale digitised features including roads (grey) and river (blue). (B) Example path produced using the A* algorithm and land classification between arbitrary points. Arbitrary points were used to emphasise how the algorithm diverts the path around a prominent obstacle; in this case, the river itself (after [37]). Produced using Landsat 7 imagery from USGS and Bing Aerial Imagery.

A land classification layer was produced initially using Landsat 7 satellite sensor imagery at 30 m spatial resolution, using four classes: bare-land, crops, bush/forest and built environment. This classification was subsequently converted to an 11 m spatial resolution, so that fine-scale features could be added to the classification, such as roads and rivers (Fig 2A). The locations of known resources (e.g. schools, markets and boreholes) were digitised at this scale using GPS coordinates from fieldwork and census data, together with village locations, to provide home and target locations for pathfinding input. An A* pathfinding algorithm was used to produce plausible human paths between villages and resources [37] (an example path can be seen in Fig 2B). Further information on the collection of routine data can be found in the supplementary material of [37].

**Fig 3:**
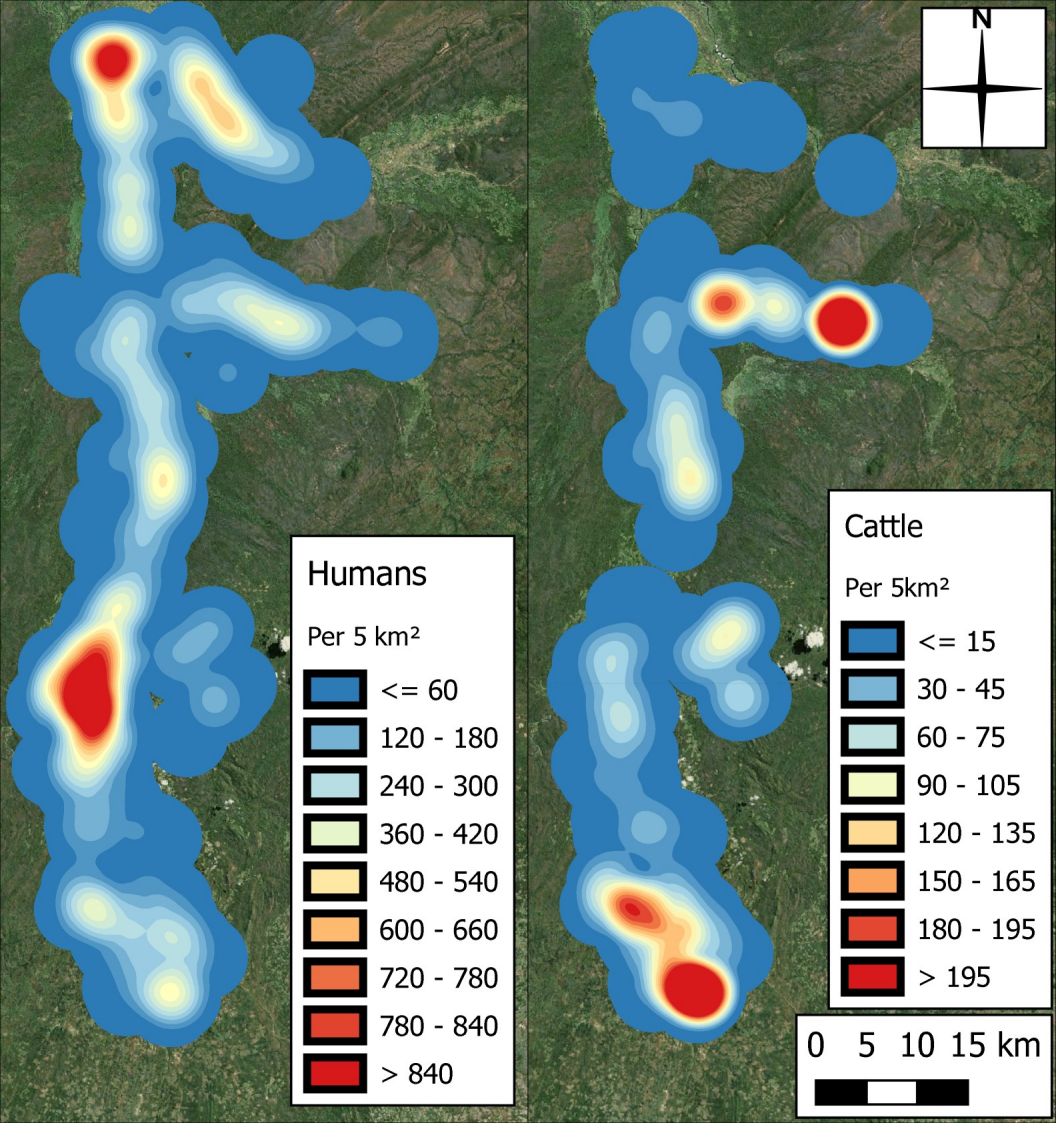
Spatial distribution of human and cattle populations in the study area, identified in the census (produced using Landsat 7 imagery from USGS).

The distributions of human and cattle populations identified in the census are presented in Fig 3. The populations are shown to be unevenly distributed, with clusters of higher human population in the central region and in the north near the airport, and higher cattle population in the east, and south towards the plateau.

**Fig 4:**
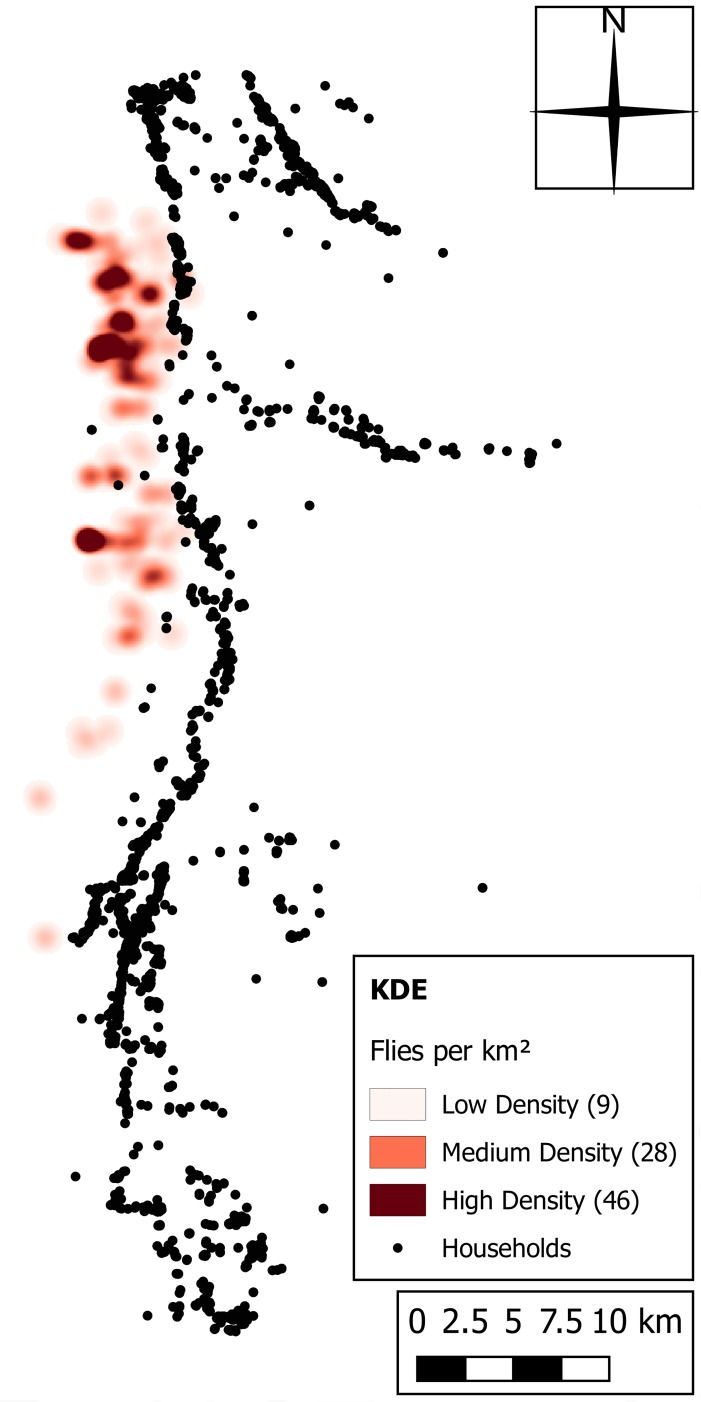
Estimation of tsetse distribution in the study area, using kernel density estimation (KDE). Numbers in brackets represent estimated values based on the number of caught flies, prior to scaling-up to account for an estimation of total population size. Reproduced from [38].

The tsetse population for all scenarios was estimated based on two tsetse surveys undertaken in 2013 (data presented in [38]), by comparing the total area covered by each individual fly round zone, whether tsetse were caught or not, with the total study area. The study area was 15 times larger than the transect area and it was assumed that for every tsetse caught 14 were missed such as to estimate a total tsetse population of 5,250 flies. The tsetse density and distribution was estimated from the sample transect data using a kernel density technique to generalise the point data. Specifically, kernel density estimation (KDE) was used to account for movement patterns and absences, reasoning that each caught fly could have started the day 800 m away from the catch site in any direction [13]. The KDE heat map output (Fig 4) shows higher densities of tsetse distributed in the north-west of the study area closer to the South Luangwa National Park, with small, but non-zero values stretching further south.

The heat map in Fig 4 highlights a disconnect between the areas of high densities of the tsetse and human populations in the area (Fig 3). This disconnect is unsurprising given the lack of tsetse habitat in these more developed regions, but also that high human densities have been linked with both tsetse decline (more than 75 humans per km^2^) and the eventual disappearing of tsetse in a region (more than 195 humans per km^2^) [29][28].

As it is difficult to estimate the size and distribution of an increasing population, four plausible scenarios were created, using the area of land considered to have anthropogenic land uses to estimate the size of increase, and the current distribution of the populations to dictate the demography and distribution of the increasing population.

### Size of population increases

With limited understanding of the predicted population increase in the study area in the future, a set of abstract population growth scenarios was selected, with a maximum population increase of approximately 60% in the final scenario compared with the original census data. To define the size of population increase, a sequentially increasing single pixel (11 m) spatial buffer was applied to all pixels categorised as crops or cultivated land, which neighbour areas of bush in the land classification. The buffer pixels classified previously as bush were transformed to crops or cultivated land to simulate an increasing region of development in the area, and the associated removal of bush (potentially suitable habitat for tsetse). The ratio of human population to developed land area was maintained as the buffer increased and is shown in Table 1.

**Table 1:** Additional human population required per scenario.

	Total Human Population	Crop Area (km^2^)	Additional Crop Area Compared with Control (km^2^)	Additional Human Population Compared with Control
Control	16024	121	0	0
1	18376	139	18	2352
2	20767	157	36	4743
3	23193	175	54	7169
4	25670	194	73	9646

### Distribution of increasing population

**Fig 5:**
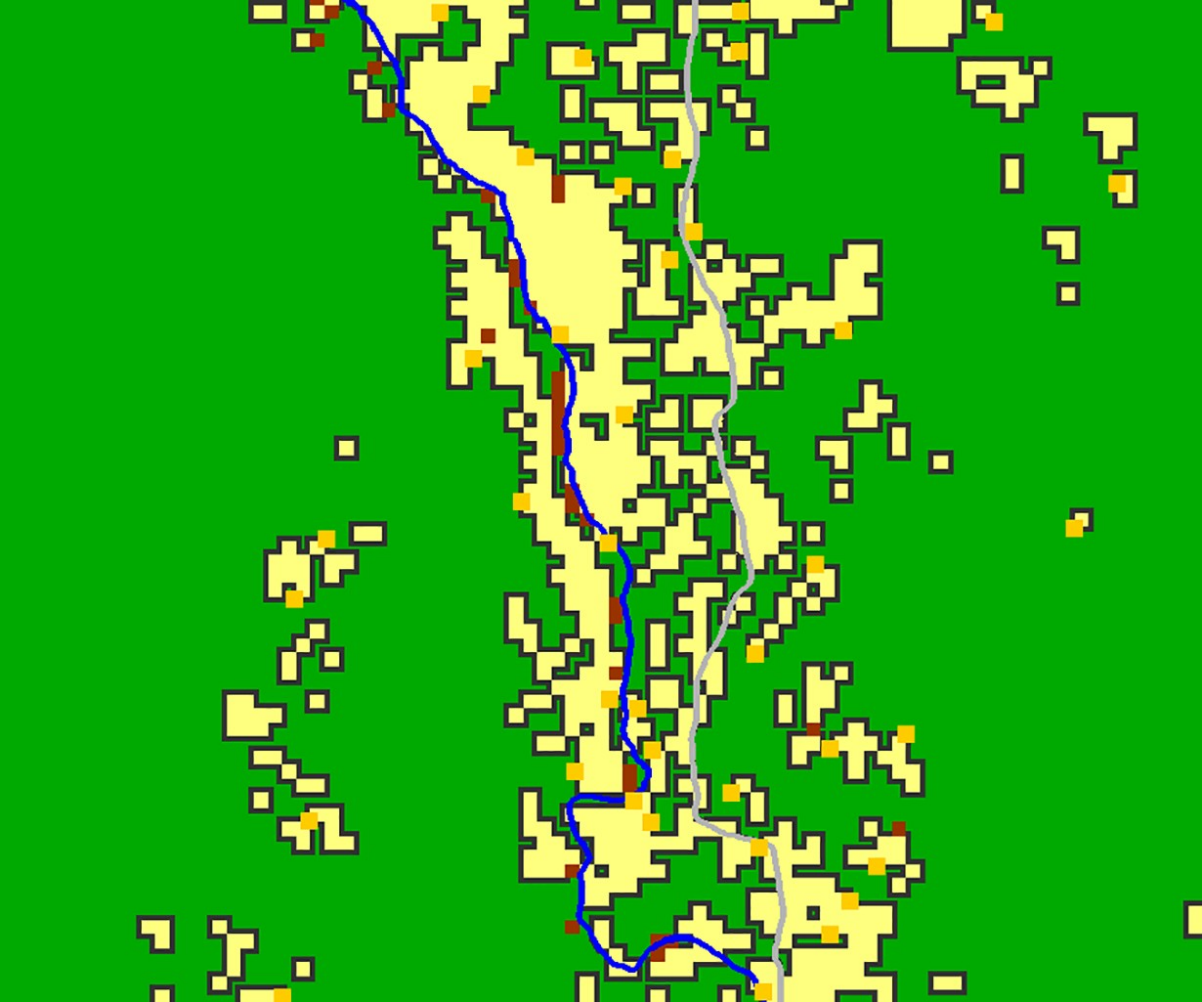
A section of the edited version of the land classification, highlighting the buffer (dark grey) used to simulate developed land encroaching on bush areas, and the location of new villages (gold) in the region.

The distribution of the current human population was used to control how the additional population would be added in the future scenarios. Until the required population for a scenario was met (Table 1), a pixel was chosen at random from the newly developed land to be the potential centre of a new village settlement. The make-up of the new village was defined by selecting a village at random from all other villages within a 500 m radius and the human and domestic animal population was cloned, acquiring information relating to agent numbers, gender, age, tribe, and type of animals kept. If there was no village within a 500 m radius of the chosen pixel, another pixel was chosen at random and the process was repeated. Populating new villages with the use of a buffer in this way ensures that any local traits in village population size and demographics are maintained, while limiting large amounts of expansion in regions which are currently very sparsely populated. An example section of the land classification with buffer and new settlements is displayed in Fig 5.

By using a technique where population increases are defined by the local population rather than a uniform probability of increasing settlement across the region, any spatial trends in cattle ownership and ethnicity are maintained, and a process which has the potential to introduce artificially new populations of humans and cattle into areas which are inhospitable in reality is avoided. For example, regions that are encroaching on large expanses of tsetse-inhabited bush do not reach the densities where fly decline is expected (e.g. 15–39 people per km^2^ [28,29]), and areas characterised by much higher human density remain further away from the tsetse. Since the focus of this study was to observe disease patterns in response to human population growth, and not tsetse decline, the increases in host agent were kept relatively small, avoiding the need to extrapolate too far into the future when, for example, climate changes may impact on tsetse populations, but also when an estimate of land-use change would be more speculative, and potentially invasive on current tsetse populations.

**Fig 6:**
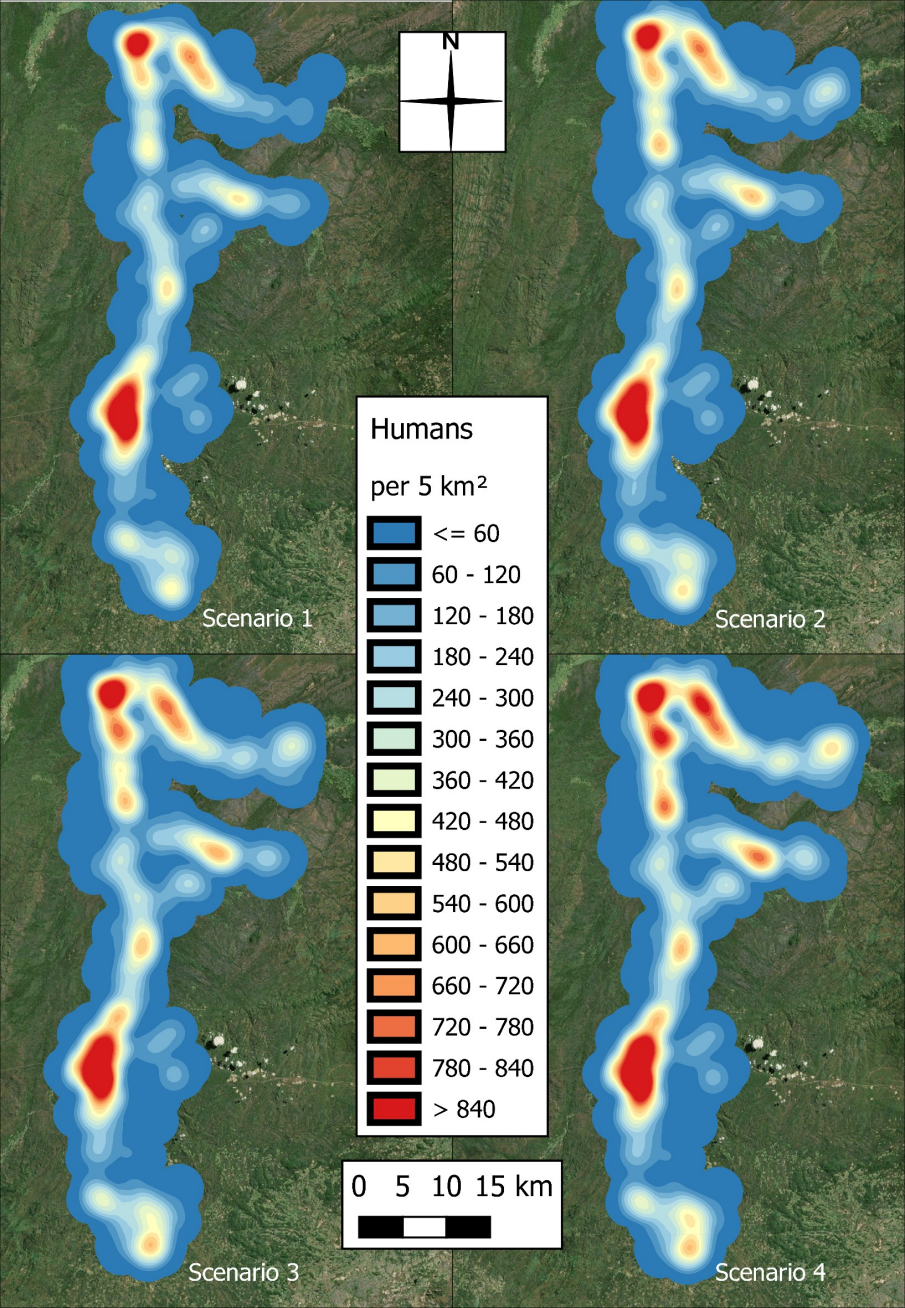
Spatial distribution of the increasing human population for scenarios 1–4 (produced using Landsat 7 imagery from USGS).

**Fig 7:**
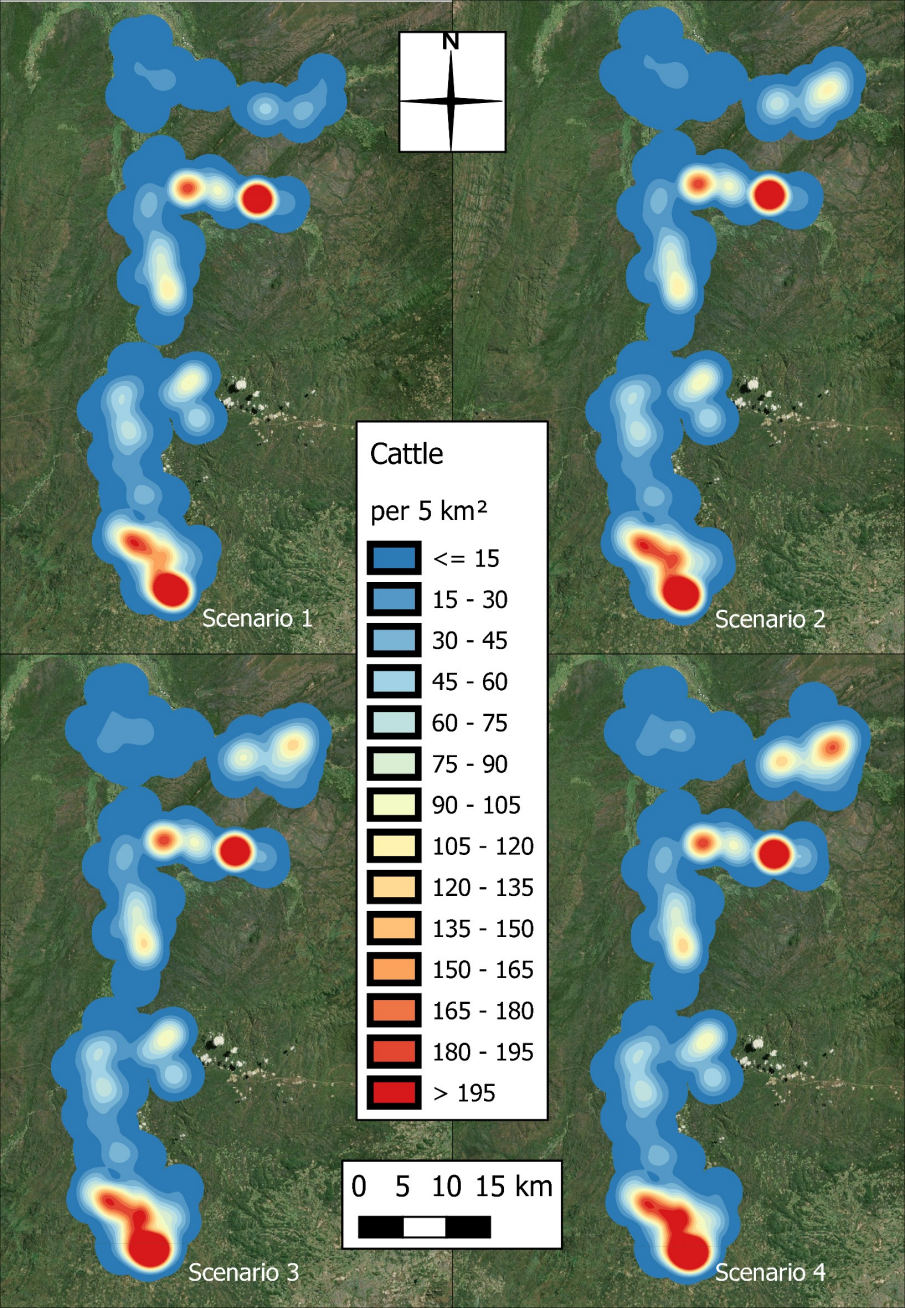
Spatial distribution of the increasing cattle population for scenarios 1–4 (produced using Landsat 7 imagery from USGS).

The distributions of humans and cattle for each scenario are illustrated in Figs 6 and 7.

**Fig 8:**
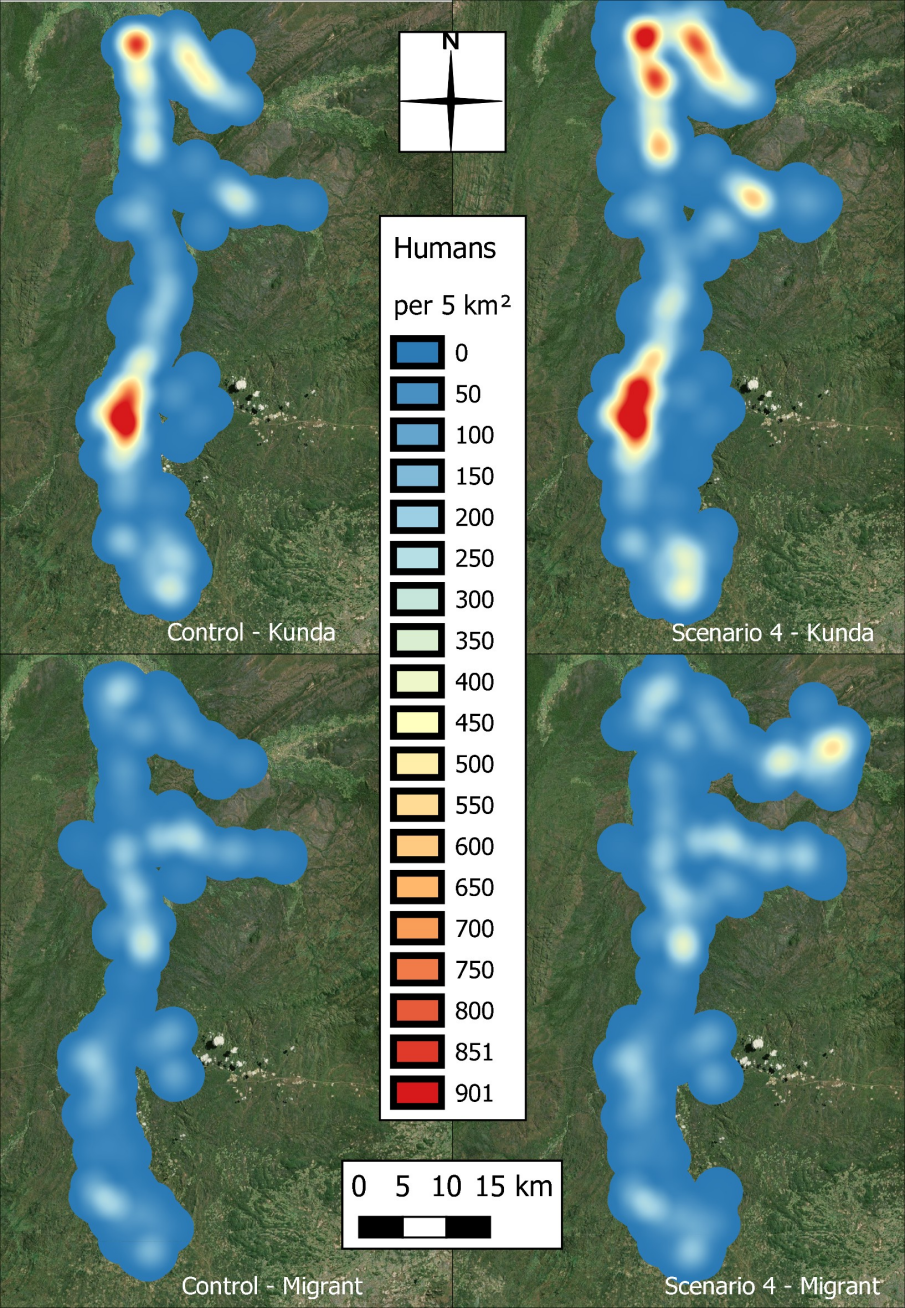
Spatial distribution of the increasing population of historically indigenous and migrant tribes in the study area, between the current population levels, and those in the most extreme scenario–scenario 4 (produced using Landsat 7 imagery from USGS).

Fig 8 shows the spatial distributions of the human populations, split by indigenous Kunda tribes and migrant tribes for the control and largest population increase scenario. In the control, the migrant population is evenly distributed through the region, however, by scenario 4 signs of dispersal are observable primarily in the north-east, but also to the north-west and east of the study area.

### Model development

The model used in this study is the most recent and advanced version of the ABM of Alderton et al. [38,39], in which seasonal climate changes were incorporated as key components of the tsetse environment, and drivers of the tsetse population life cycle. This section outlines the relevant modifications required for the present study. The ABM’s behaviour itself was not adapted for this research from that applied to produce the baseline case, and the same initial tsetse population input is used for each scenario. By not changing the behaviour of the model, any changes observed in the system can be attributed directly to the host population increases and altered tsetse habitat.

As in previous work, paths between home and resource were produced using the A* pathfinding algorithm [37]. Four agent types were included in the ABM, together with an areal representation of wildlife which is mapped to areas classified as bush only. As a result, the availability of wildlife feeds for tsetse, along with resting sites, is modified with the development of bush area between scenarios. Human, cattle, other domestic animals and tsetse used in the ABM were constructed as four separate classes, with populations modelled 1:1 with the population in each scenario. Each simulation was initialised with the full host agent population of the relevant scenario, and the stable tsetse population presented in the baseline model. Each class had its own initial information and storage structures for events that occurred through the simulation. The ABM was written in Python 2.7 using an object-oriented framework, and run on the Lancaster University High End Computing (HEC) Cluster, with all spatial data being processed using Quantum GIS 1.8.0.

To allow the model to initialise and stabilise, the simulation was run for a year, allowing a ‘burn-in’ period, before the results for this paper were produced. The results presented below represent years two to four of the simulation. One hundred repeat simulations were used to produce the results presented.

## Results

### Control results summary

**Fig 9:**
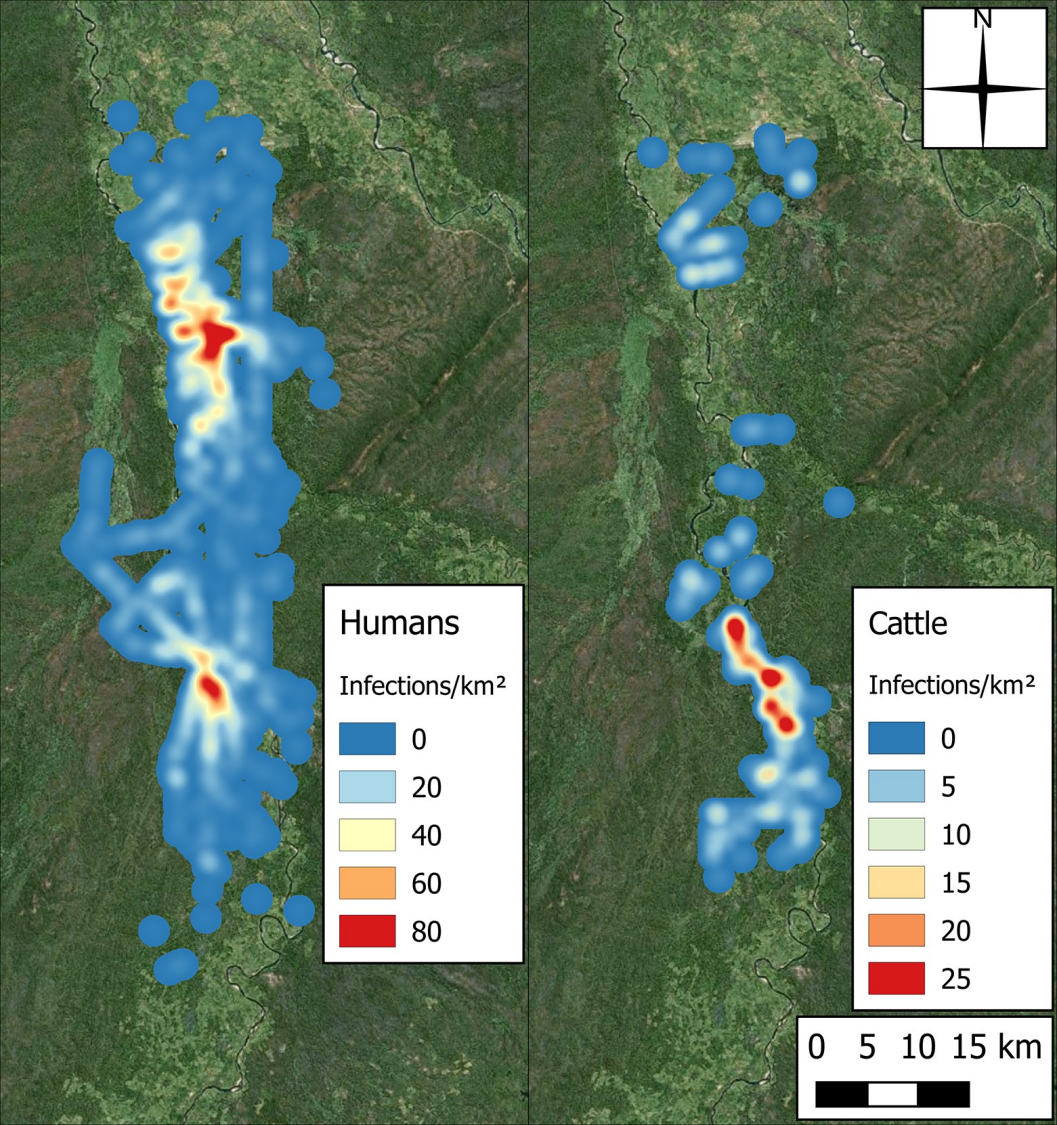
Heat surface of the region representing aggregate number of infections (Left: Human, Right: Cattle) across the 100 repeats of the control simulations, with pixel values taking the units of infections per square kilometre (produced using Landsat 7 imagery from USGS).

Across the three year control simulation, the approximate incidence rate for human and cattle rHAT infections was 0.355 per 1000 person-years (SE = 0.013) and 0.281 per 1000 cattle-years (SE = 0.025). Fig 9 shows how these infections were grouped spatially as a heat surface, presenting the aggregate number of infections across both years and the 100 repeats. In addition to the difference in number of infections, there is a clear spatial difference between the plots for humans and cattle with a hotspot in human infections existing where no cattle infections occur; a region of low cattle population. The human infections are fairly evenly distributed throughout the region, while most cattle infections occur further south.

### Population infection rates

**Fig 10:**
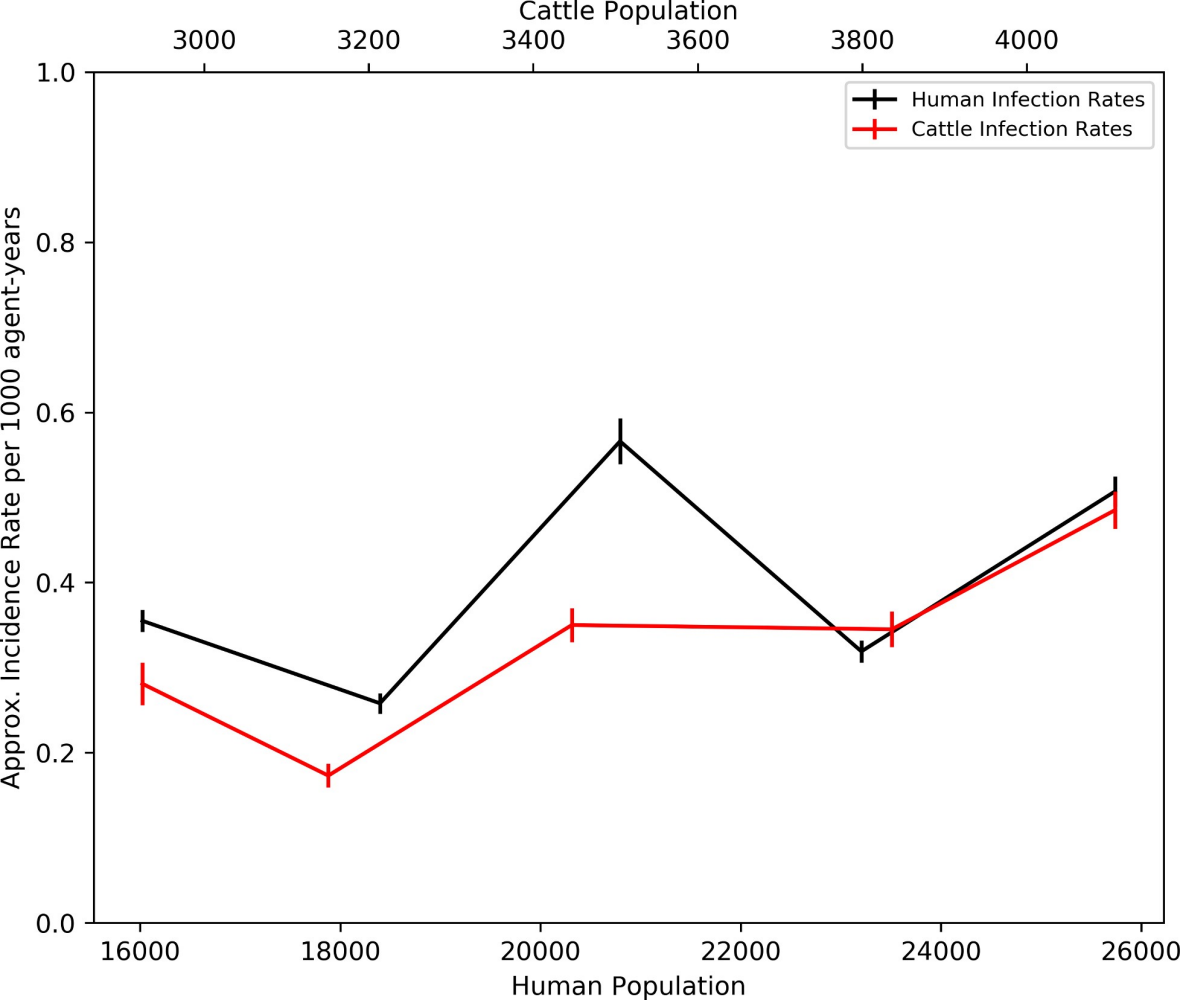
Mean approximate incidence rates (per 1000 agent-years) for human and cattle agents, plotted against population sizes.

Increasing the human and cattle population size resulted in a general increase in incidence rates for both agent types (Fig 10). The first iteration of population increase (Scenario 1) shows a slight decrease in incidence rates for both human and cattle followed by a sharp increase to the population sizes of the second scenario. After the sharp increase, the incidence rates in cattle stabilise at approximately 0.35 per 1000 cattle-years for scenarios 2 to 3, before increasing to 0.5 in scenario 4. The human incidence rates are unstable between scenarios 2 to 4, peaking at an incidence of approximately 0.6 per 1000 human-years for scenario 2, and 0.3 in scenario 3, a level below that observed in the control simulation.

**Fig 11:**
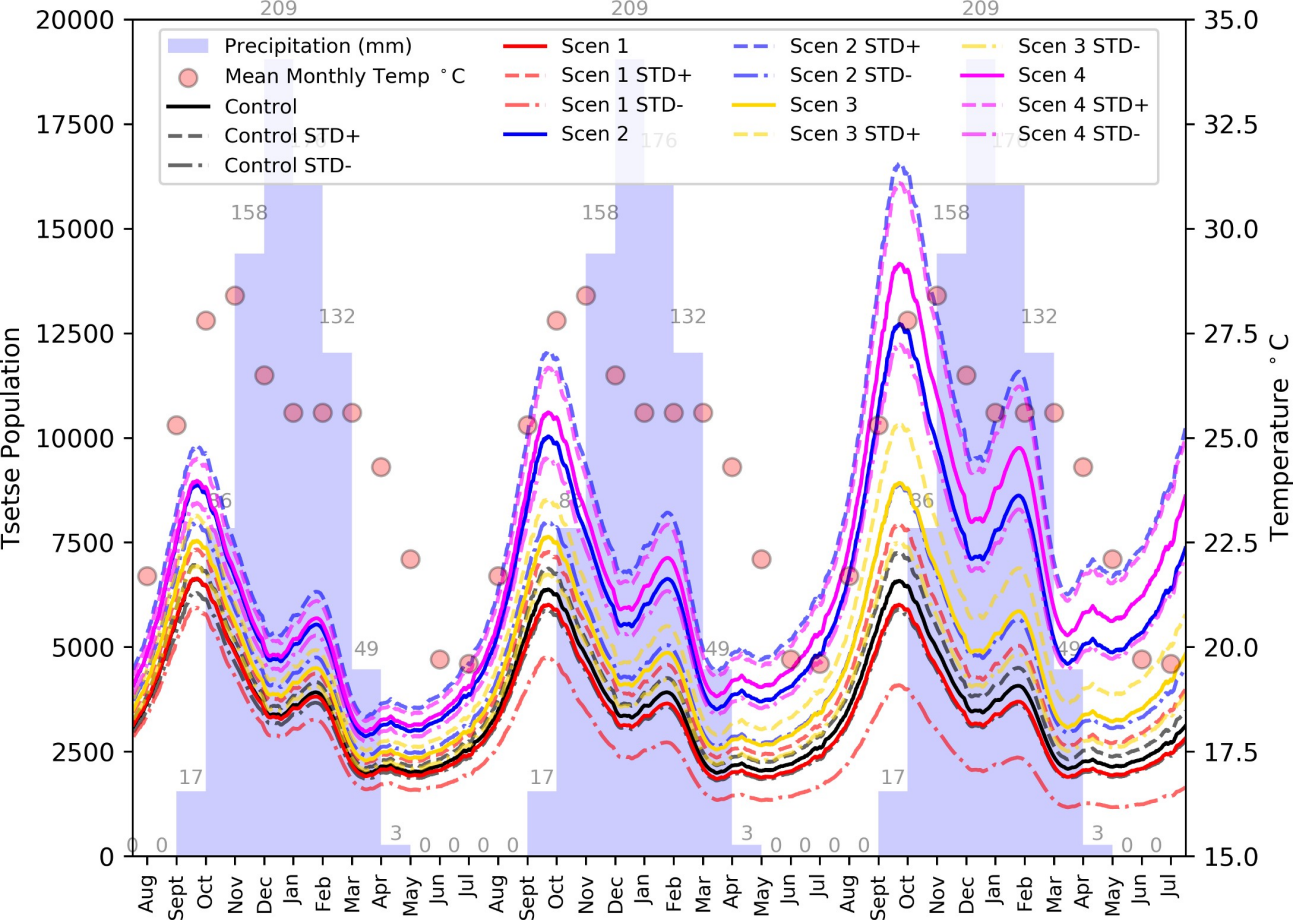
Mean daily population size of adult tsetse flies by sex (+/- standard error) for all scenarios, overlain on mean monthly temperature and precipitation for the three-year simulation period.

Fig 11 plots the variation in the adult tsetse population size through the three year simulation period for all scenarios. Scenario 1 produces a similar trend in population size throughout the simulation compared with the control. However, scenarios 2 to 4 display a varying degree of growth, with the mean end populations ranging between approximately 5,000 tsetse for scenario 3, to 8,500 for scenario 4. Perhaps surprisingly, given a lower potential host population, scenario 2 consistently presents a larger tsetse population than scenario 3. However, the tsetse population for scenario 2 is within one standard deviation of the populations for both scenarios 3 and 4 and, therefore, is not significantly different.

### Spatial infection

**Fig 12:**
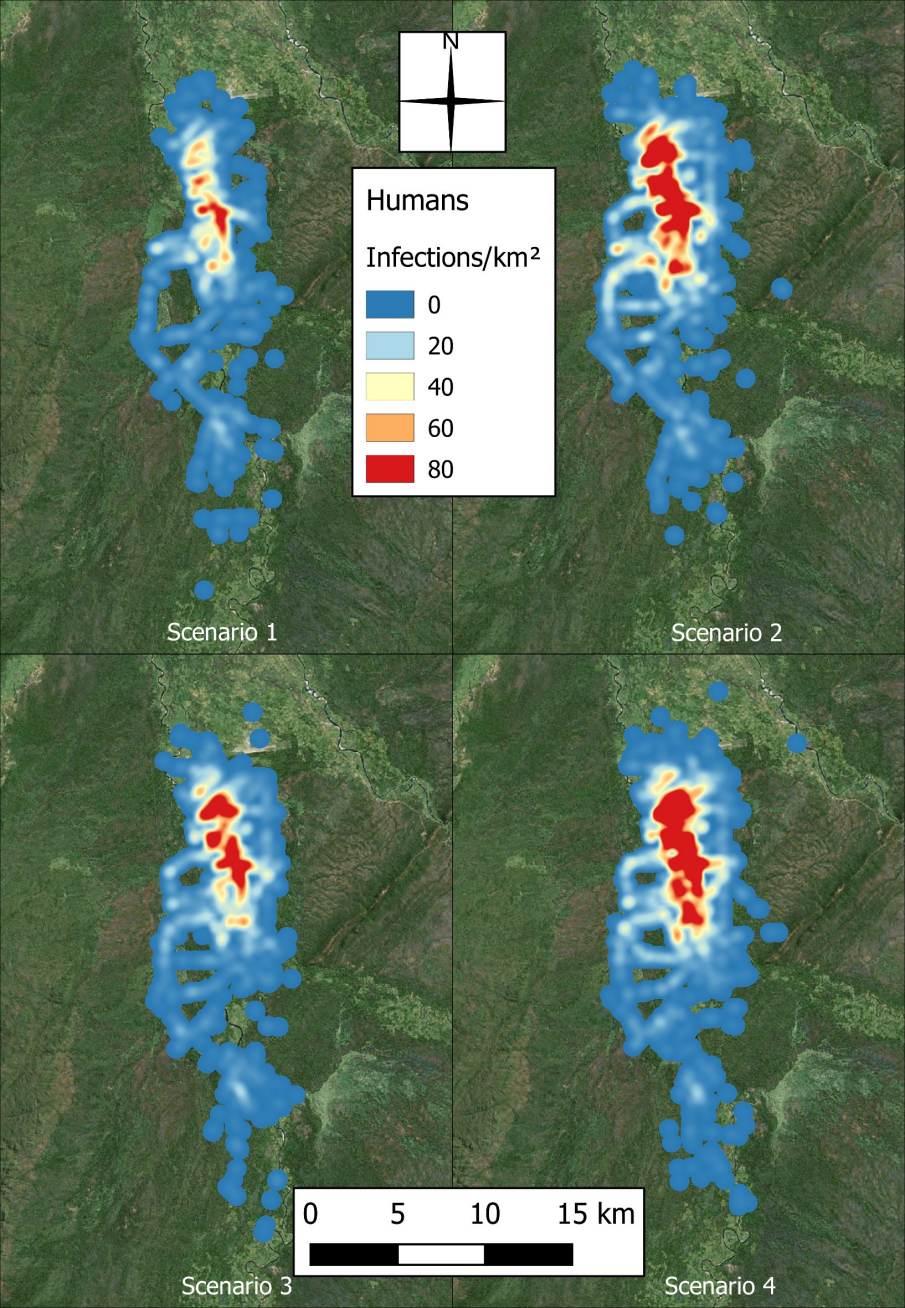
Heat surfaces representing the aggregate number of human infections across the 100 repeats, with pixel values taking the units of infections per square kilometre (produced using Landsat 7 imagery from USGS).

**Fig 13:**
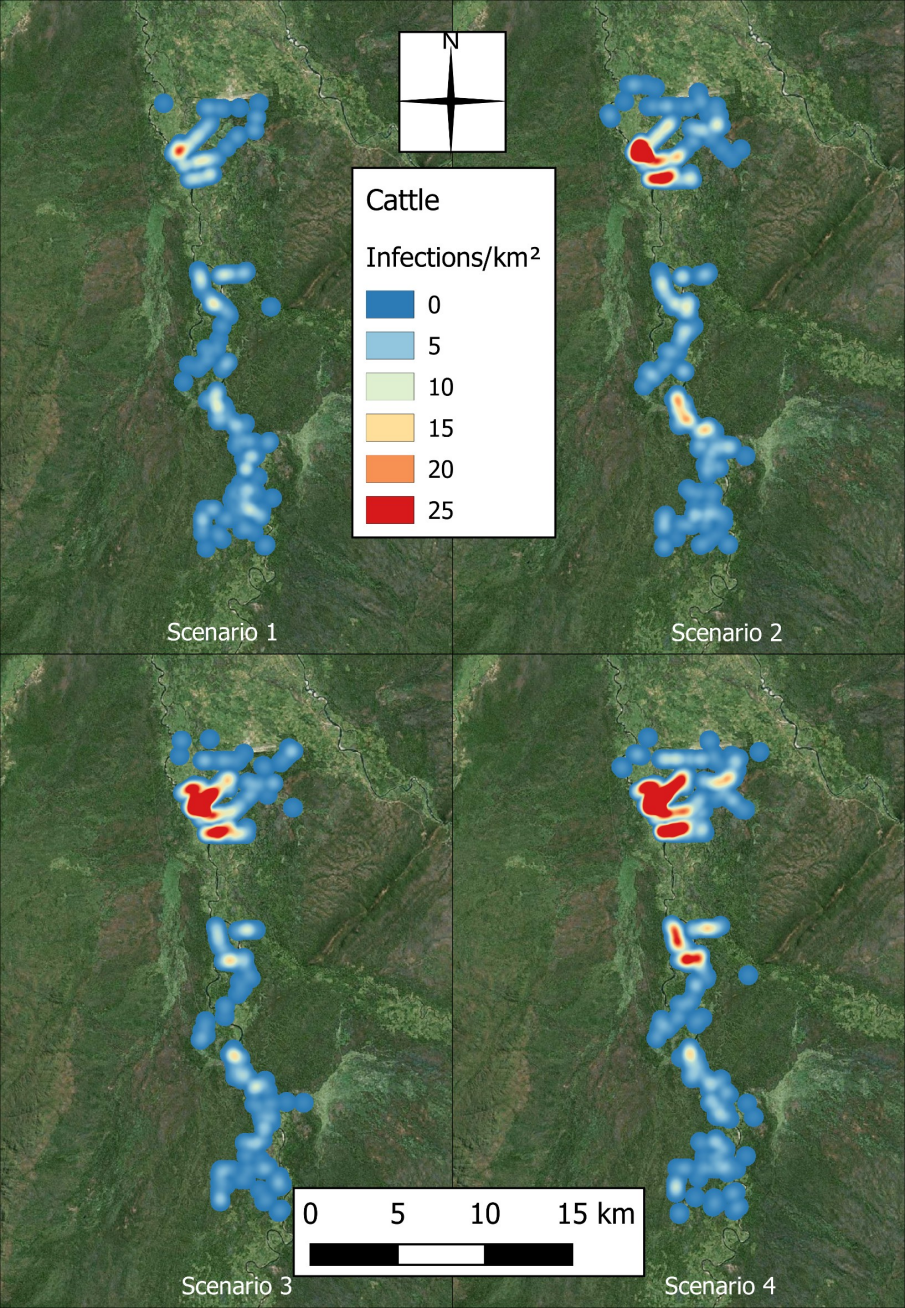
Heat surfaces representing the aggregate number of cattle infections across the 100 repeats, with pixel values taking the units of infections per square kilometre (produced using Landsat 7 imagery from USGS).

Variation in the spatial extent and distribution of agent infections was observed in the simulation as the populations were increased. Figs 12 and 13 map the aggregate human and cattle infections, respectively, for each scenario. Both figures show a greater dispersal of infections as the populations increase, and while the hotspot of human infections moves slightly further north and becomes more concentrated, additional hotspots appear in the cattle infection distribution towards the centre and south of the region.

### Demographic distribution of infection

The control simulation displayed a high proportion of human infections occurring in agents while travelling long distances to and from school (50%), and an increase in population shows a reduction in this percentage to only 7.9% in scenario 4. Table 2 shows that as the largest populations are reached, the highest proportion of infections changes from the longer distance resource trips to the shorter distance trips of collecting water (46.6%) and firewood (19.1%).

**Table 2:** Average proportion of human infections attributable to each activity, for the control and each scenario, represented as a percentage.

	Control		Scenario 1		Scenario 2		Scenario 3		Scenario 4	
State	*Average*	*SE*	*Average*	*SE*	*Average*	*SE*	*Average*	*SE*	*Average*	*SE*
*School*	**50**	**12.5**	**23.9**	**12.7**	**15.8**	**7.8**	**12.8**	0.9	**7.9**	0.4
*Resting*	**3.2**	**3.4**	**6.2**	**5.6**	**6.9**	**4.0**	**7.9**	0.4	**8.0**	0.3
*Market*	**0.1**	**0.7**	**1.8**	**3.5**	**0.9**	**1.7**	**0.6**	0.1	**0.3**	0.1
*Firewood*	**5.8**	**5.0**	**8.2**	**7.7**	**12.1**	**4.9**	**20.9**	0.8	**19.1**	0.6
*Farm*	**15.3**	**7.8**	**20.3**	**10.4**	**19.4**	**6.8**	**18.8**	0.9	**18.1**	0.6
*Water*	**25.5**	**11.9**	**39.7**	**15.4**	**45**	**10.3**	**39.0**	1.0	**46.6**	0.8
*Graze Cattle*	**0**	**0**	**0**	**0**	**0**	**0**	**0**	0	**0**	0
*Water Cattle*	**0**	**0**	**0**	**0**	**0**	**0**	**0**	0	**0**	0

The age groups which contain school-aged children (5–10, 10–18) show the greatest decrease in approximate incidence rates between the control and scenario 4 (Table 3), with only scenario 2 confounding the trend. Conversely, adult human agents (ages 18–60, 60+) show increasing incidence rates through the scenarios, reaching a peak of 0.966 and 0.967 per 1000 human-years in the 18–60 and over 60 age categories, respectively, in scenario 4.

**Table 3:** Approximate incidence rates of different age groups, for each scenario.

	Control		Scenario 1		Scenario 2		Scenario 3		Scenario 4	
* *	*Average*	*SE*	*Average*	*SE*	*Average*	*SE*	*Average*	*SE*	*Average*	*SE*
**Age**										
age1-5	**0.024**	0.004	**0.021**	0.003	**0.039**	0.004	**0.025**	0.003	**0.028**	0.003
age5-10	**0.699**	0.030	**0.320**	0.020	**0.576**	0.038	**0.177**	0.011	**0.235**	0.014
age10-18	**0.645**	0.028	**0.308**	0.019	**0.547**	0.035	**0.180**	0.012	**0.238**	0.012
age18-60	**0.239**	0.012	**0.308**	0.018	**0.672**	0.041	**0.580**	0.025	**0.966**	0.036
age60+	**0.185**	0.025	**0.365**	0.030	**0.811**	0.057	**0.538**	0.035	**0.967**	0.042

Results for the control show similar incidence rates between genders and for cattle owners and those without cattle (Table 4). However, from scenario 1 onwards, a greater incidence rate is observed in simulated female agents than males, and for scenario 4, the approximate incidence rate for those in households without cattle is 0.527 per 1000 human-years, a notable increase compared to the control.

**Table 4:** Approximate incidence rates of different sexes and cattle ownership, for each scenario.

	Control		Scenario 1		Scenario 2		Scenario 3		Scenario 4	
* *	*Average*	*SE*	*Average*	*SE*	*Average*	*SE*	*Average*	*SE*	*Average*	*SE*
**Gender**										
Male	**0.362**	0.015	**0.200**	0.010	**0.426**	0.020	**0.274**	0.012	**0.429**	0.016
Female	**0.348**	0.015	**0.313**	0.017	**0.703**	0.036	**0.363**	0.016	**0.583**	0.021
**Cattle House**	** **		** **		** **		** **		** **	
TRUE	**0.398**	0.025	**0.321**	0.020	**0.486**	0.025	**0.357**	0.021	**0.422**	0.019
FALSE	**0.346**	0.013	**0.244**	0.013	**0.585**	0.029	**0.310**	0.013	**0.527**	0.019

Table 5 suggests that the difference in infection rate between the indigenous Kunda tribe and the other, immigrant tribes which is observed in the control simulation, becomes less pronounced as the population sizes increase through the scenarios.

**Table 5:** Approximate incidence rates by immigrant/non-immigrant tribes, for each scenario.

	Control		Scenario 1		Scenario 2		Scenario 3		Scenario 4	
* *	*Average*	*SE*	*Average*	*SE*	*Average*	*SE*	*Average*	*SE*	*Average*	*SE*
Ethnicity										
Kunda	**0.245**	0.011	**0.222**	0.012	**0.532**	0.029	**0.275**	0.014	**0.482**	0.018
Immigrant	**0.622**	0.028	**0.341**	0.018	**0.645**	0.028	**0.417**	0.017	**0.559**	0.021

## Discussion

### General observations

The results and trends presented as outputs from the simulation are conditional upon a wide range of inputs and parameters across a complex system, each set with varying degrees of confidence. As such all results relate to the model, and only to the local reality on the ground, to the extent that the model is a close representation of local conditions. The objective was to construct a *plausible* model of reality such that experiments could be conducted in the constructed “universe”. The results of such experiments then necessarily relate to the model structure, parameters and conditions of that constructed universe. As a result, model output should be interpreted in terms of general patterns, trends and changes across the scenarios.

The approximate incidence rates for both cattle and human rHAT infections exhibit an increase between the control and the maximum population tested (scenario 4). However, the pattern observed between scenarios for each agent type is different, with a steady increase observed in the modelled cattle populations, and fluctuations in the human population. This highlights the complex relationship between vector-host contact network, feed acquisition and infection reservoir. In scenario 1, infection rate is reduced in both hosts. Although this could be attributable to a slight increase in developed ‘buffer’ around the majority of the settled area, it is likely to be a response to the way that the population increase is evenly distributed across the study area and, therefore, many of the new agents will have low exposure to tsetse risk.

For scenario 2, the increase in cattle infection rate suggests greater connectivity between vector and the primary source of tsetse feeds than experienced in scenario 1. This availability leads to an increase in tsetse population which, in turn, increases the mobility of the parasite, and exposure of the similarly increasing human population. This is exhibited by the doubling infection rate observed in the simulation for non-cattle owning households. The plateau in cattle incidence rates for scenario 3 suggests that a threshold has been reached, such that the addition of uninfected hosts to the system is *impeding* the transmission cycle of rHAT. Tsetse flies are more susceptible to acquiring a midgut infection on their first feed [49]. Consequently, with an increasing host population not exhibiting an rHAT infection, the rate of uptake of the disease by tsetse will have been reduced. An additional factor is the apparent decrease in tsetse population when compared to scenarios 2 and 4 (Fig 11), which must be attributable to the relative disconnect between vector and host given that there is no parametric increase in tsetse mortality with increasing size of developed area. For example, compared to scenario 2, the infection rate among school-age children is much reduced. School trips are associated with long walks and, therefore, greater exposure. For these walks to now produce fewer infections, a reduced level of exposure must be present, such as the modification of nearby tsetse habitat into human-developed land. Although it is noted that the developed land increases were small, it could be enough to reduce the number of contacts. For example, while the maximum displacement of a simulated tsetse fly is 800 m from the agent’s daily start location, movements within that buffer are random and, therefore, unlikely to reach this linear limit within an activity period. Furthermore, as the scent detection range of the tsetse is limited to 140 m, small increases in the distance between host homes (and pathways to resources) and tsetse habitat could be significant.

By scenario 4, the reduction in connectivity is countered by the growth of more spatially marginal populations, living closer to the tsetse habitat, and further away from important resources such as river water. This pattern returns a peak in both human and cattle infection rates for the study, but also peaks in tsetse population, and proportion of infections acquired when collecting water supplies and firewood. These trips are associated with more frequent collections which affect the whole demographic, a change which is mirrored in both the incidence rates of adults (age groups over 18) and female agents, who are more likely to be assigned routines associated with domestic resource collection than, for example, cattle herding.

### Ethnicity of infection

It is initially surprising that immigrant incidence rates do not increase through the scenarios in comparison to the indigenous tribe, the Kunda. However, the model does not preferentially increase migrant tribe populations, as the purpose of this study was to investigate the impact of population growth throughout the study area, including increases in the indigenous population and the development of communities of recent migrants to the region. However, the results suggest that indigenous tribes are also living in close proximity to the tsetse, as the incidence rates are increasing at a similar rate to that of the migrants. This does not represent the spatial marginality of migrants that was expected, despite the method of population growth ensuring that new villages had the same demographic make-up of neighbouring villages. Although the proportion of population attributable to migrant tribes increases by 3% (from 29% to 32%) between the control and scenario 4, Fig 8 highlights that not only are migrant communities currently interwoven with those of the indigenous population, but the growth of migrant population in spatially marginal areas does not necessarily lead to an increase the population exposed to the tsetse vector. In this example, this is supported by the protrusion of a migrant population in the north-east of the study area, away from the tsetse.

### Spatial distribution of infection

The distribution of human infections becomes increasingly concentrated in the northern section of the transect, mirroring the spatial distribution of cattle infections in the region–an area shown to have limited previous exposure to cattle. The increase in population in this region occurs as it is already a populous area, being close to the river and the airport. The progressive increase in cattle population here (observed in Fig 7) coincides with peak cattle infections in the region and, therefore, is a prominent reservoir for infection adjacent to the tsetse zone (Fig 4). Although cattle ownership appears to reduce the likelihood of infection (Table 4), there could be negative outcomes for neighbouring villagers.

### Wider implications

This research has highlighted the significance of connectivity in studies of vector-borne diseases, through demonstrating the changes in simulated infection rates in both human and cattle populations with population growth. For example, it has been shown that an increase in population does not necessarily mean an increase in infection rate, meaning there must be other factors impacting on the system, such as connectivity. This potentially highlights the importance of modelling at an individual level, and incorporating fine resolution spatial variability. This importance of connectivity is also demonstrated by the fact that there is not a linear relationship between the tsetse population and human/cattle population increase. Furthermore, in-migration into a region with a growing population is not necessarily a causal factor in increasing infections rates, as these populations need not be preferentially exposed to the risk factors associated with the disease. Indeed, any alteration of transmission pattern is a product of a complex relationship between demography, geography and biology which must be explored where suitable, local-scale mitigation strategies are required. The model used in this study has allowed this demographic differentiation, which would otherwise be impossible using more traditional modelling techniques.

Observation of the changes in the trend in incidence rates by demographic group highlights that resource provision alongside population growth may be just as significant as managing the growing population and its distribution in the region in future. Results suggest children under 18 have decreasing incidence rates as the population grows, while the incidence rate may increase in adults–a change which could have a negative effect on economic productivity in the future. This behaviour is reflected in the proportion of infections that occur during different activities, with the shorter, more frequent trips such as water and firewood collection capturing an increasingly larger proportion of the infections, and longer, less frequent trips such as travelling to school receiving a lower proportion as the population increases. This trend suggests that promoting well-targeted resource provision (e.g., schools, clinics) could be an important factor in mitigating the impacts of a growing population.

### Benefits of modelling techniques

The ABM could be useful for formulating policy and for local decision-makers, with the model generating data suggesting who (e.g., by age, by gender, by tribe) might be most at risk of infection in the future, and where. The model draws attention to the importance of connectivity between hosts and vectors, but also how an increase in host population should not simply be seen as an increasing reservoir, since under certain circumstances it can also reduce the rate at which the pathogen is transmitted through the landscape.

The results presented in this paper update our current understanding of tsetse fly dynamics and the rHAT disease transmission system. The ABM approach is suitable for use by local decision-makers (e.g., in the Luangwa Valley) to investigate alternative mitigation strategies (e.g., changes in human behaviour through education; vector control strategies such as insecticide application; relocation of resources such as schools). Most importantly, the individual level model presented here, which includes seasonally varying effects on tsetse populations, provides a suitable framework with which to model the impacts of many different future scenarios (e.g., land cover and land use changes; changes in human, livestock or tsetse population distribution; changes in climate) in the Luangwa Valley (and elsewhere if re-fitted locally).

The observed model outputs presented here cannot be created without using the ABM approach (e.g., cannot be created using a population-level, partial differential equation model), and so may be regarded as novel in terms of rHAT transmission dynamics. Specifically, interrogation of the impacts of a wide variety of interventions and changes in system drivers is only possible through the proposed ABM modelling framework because the connection between the various agents and the geographical and temporal environments on which their lives are played out is captured explicitly. This allows the geographical environment and agents’ initial conditions to *constrain* and determine the dynamic contact network between the multiple agent classes (segmented by age, gender, tribe etc.), which ultimately is the “funnel” through which transmission must occur. The ABM also spatially references each contact event in this dynamic contact network such that the realised transmission events between agents have a dynamic, spatially explicit mapping. In this paper, we show that this spatially explicit, as well as demographically rich, information is invaluable to both our understanding of the disease transmission process in specific contexts, but also to decision-makers who aim to reduce or eradicate disease.

## Conclusion

In this research, a very fine spatial resolution ABM was used to investigate the possible future impact of an increase in human and animal host population on rHAT transmission in a case study in Eastern Province, Zambia. The investigation showed that as populations increase, the level of spatial connectivity between tsetse vector and human and domestic animal hosts increases as people are forced to occupy increasingly marginal spaces. Importantly, we found that this led to an increase in the proportion of infections associated with shorter trips, as new settlements develop further from pre-existing water resources. This reflects a shift away from children being the most at risk in the control study, towards agricultural workers being most exposed which, if realised, would have implications for economic productivity in the region. The non-linear relationships observed between host population growth and both the size of the tsetse population, and the overall incidence rates, highlights the complexity of the relationship between the occupation of space in the model, and the infectious state of interacting agents, and how this relationship reacts to a modification of the population. Future research will focus on testing the applicability of this modelling framework to similar sites in other affected regions such as Zimbabwe.
